# Modulation in current density of metal/n-SiC contact by inserting Al_2_O_3_ interfacial layer

**DOI:** 10.1186/1556-276X-8-116

**Published:** 2013-03-02

**Authors:** Shan Zheng, Qing-Qing Sun, Wen Yang, Peng Zhou, Hong-Liang Lu, David Wei Zhang

**Affiliations:** 1 State Key Laboratory of ASIC and System, Department of Microelectronics, Fudan University, Shanghai, 200433, China

**Keywords:** Contact resistance, Schottky barrier height, SiC, Atomic layer deposition

## Abstract

Metal contact to SiC is not easy to modulate since the contact can be influenced by the metal, the termination of the SiC, the doping, and the fabrication process. In this work, we introduce a method by inserting a thin Al_2_O_3_ layer between metal and SiC to solve this problem simply but effectively. The Al_2_O_3_/n-SiC interface composition was obtained with X-ray photoemission spectroscopy, and the electrical properties of subsequently deposited metal contacts were characterized by current–voltage method. We can clearly demonstrate that the insertion of Al_2_O_3_ interfacial layer can modulate the current density effectively and realize the transfer between the Schottky contact and ohmic contact.

## Background

Silicon carbide is a promising material for numerous electronic applications due to its wide bandgap, high breakdown electric field, high thermal conductivity, and high saturation velocity [1]. These excellent properties make SiC suitable for high-temperature, high-power, and high-frequency applications. For high-performance and high-frequency devices in these applications, metal/SiC contact plays very important roles. However, the traditional method for fabricating Schottky contact and ohmic contact are so different, and it will unavoidably add to the processing difficulty and cost [2].

The Schottky barrier height (SBH) is the key factor that determines whether the electrical behavior is an ohmic contact or Schottky contact: a low SBH is necessary to create a good ohmic contact, while a large SBH is required to form a good Schottky contact. According to the thermionic emission model [3], the direct reflection of the SBH is the reverse current density, and therefore, by controlling the Schottky barrier height, we can modulate the current density and acquire the needed contact type without modifying the fabrication process.

**Figure 1:**
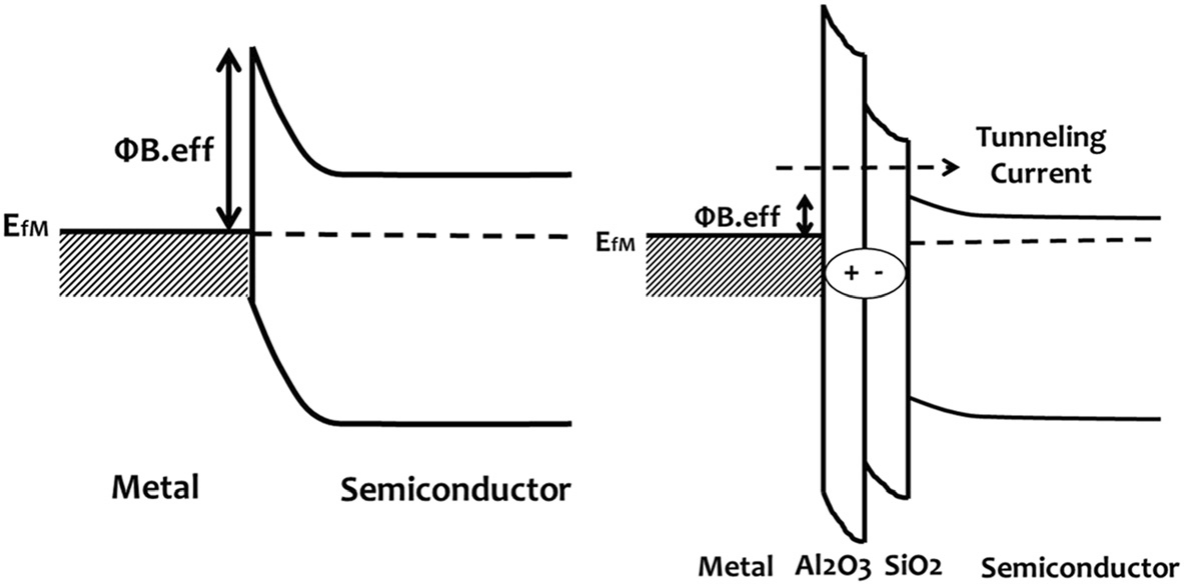
**A schematic band diagram of a shift in the metal/semiconductor’s high barrier height.** This is done by forming an electronic dielectric dipole between the insulator and the oxide of semiconductor in accordance with the bond polarization theory.

In a previous study, Connelly et al. [4] have raised a method to reduce the SBH of the metal/Si contact by using a thin Si_3_N_4_ through the creation of a dielectric dipole [5]. Similar researches have been dedicated to the study of the SBH modulation on Ge [6-9], GaAs [10], InGaAs [10,11], GaSb [12], ZnO [13], and organic material [14] by inserting different dielectrics or bilayer dielectrics. According to the bond polarization theory [15], an electronic dielectric dipole is formed between the inserted insulator and semiconductor native oxide which results in a shift of the SBH, as Figure 1 depicts. The origin of the dipole formation at the dielectric/SiO_2_ interface is described in Kita’s model [16], and in this model, the areal density difference of oxygen atoms at the dielectric/SiO_2_ interface is the driving force to form the dipole. Since the areal density of oxygen atoms (*σ*) of Al_2_O_3_ is larger than that of SiO_2_, the *σ* difference at the interface will be compensated by oxygen transfer from the higher-*σ* to the lower-*σ* oxide which creates oxygen vacancies in the higher-*σ* oxide (Al_2_O_3_) and negatively charged centers in the lower-*σ* oxide (SiO_2_), and the corresponding direction of the dipole moment is from SiO_2_ to Al_2_O_3_. As a result, this dipole is a positive dipole which can reduce the SBH and therefore increases the current density. As the thickness of the inserted insulator increases, it becomes more difficult for the current to tunnel through the insulator, and the tunneling barrier is the dominant factor of the total barrier height, which decreases the current density in the end.

In this work, we demonstrate the modulation of the current density in the metal/n-SiC contact by inserting a thin Al_2_O_3_ layer into a metal-insulator-semiconductor (MIS) structure. Al_2_O_3_ is chosen as the interfacial insulator for its large areal oxygen density (*σ*) which means that the formation of dipole is much stronger and shifts the SBH more effectively than that induced by other insulators based on the bond polarization theory [15] and Kita’s model [16]. As for the choice of metal, aluminum (Al) is suitable due to its low work function (4.06 to 4.26 eV) for the investigations of the Fermi level shift toward the conduction band of SiC (electron affinity = 3.3 eV).

The analysis of the Al_2_O_3_/SiC interface during the formation of Al_2_O_3_ was obtained with X-ray photoemission spectroscopy (XPS), and the electrical properties of Al/ Al_2_O_3_/SiC with different thicknesses of the inserted Al_2_O_3_ were characterized by current–voltage (*I*-*V*) method. Since the current density as well as the contact resistance was found to be sensitive to the Al_2_O_3_ thickness, we carefully varied the Al_2_O_3_ thickness from 0.97 to 6.3 nm and finally have acquired the experiment results that can describe the modulation of current density by changing the thickness of the insulator.

## Methods

**Figure 2:**
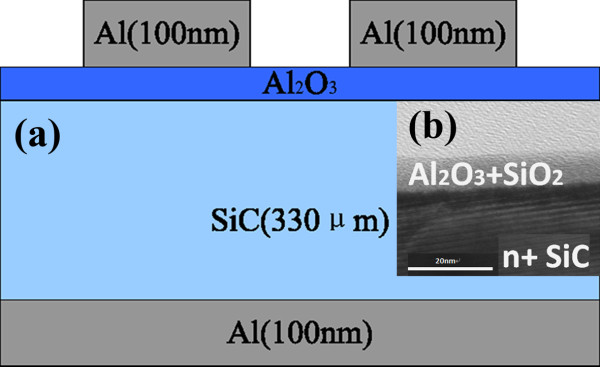
**Schematic diagram of MIS structure and cross-sectional TEM of Al/Al**_**2**_**O**_**3**_**/SiC.** (**a**) A schematic diagram of the MIS structure. (**b**) The cross-sectional TEM of the Al/Al_2_O_3_/SiC contact, showing that Al_2_O_3_ was deposited uniformly as a fully amorphous film.

We prepared an Al/Al_2_O_3_/SiC MIS structure on n-type C-terminated 6H-SiC with a carrier concentration of 1 × 10^16^ cm^−3^ epitaxially deposited by metal-organic chemical vapor deposition. Firstly, samples were cleaned in solutions of detergent, H_2_SO_4_/H_2_O (1:4), NH_4_OH/H_2_O_2_/H_2_O (1:1:5), and HCl/H_2_O_2_/H_2_O (1:1:6), and treated with HF/H_2_O (1:50) solution, followed by rinsing in deionized water to remove native oxide at the surface. Secondly, the Al_2_O_3_ film was then deposited using trimethylaluminum and H_2_O as precursors at 200°C by atomic layer deposition (ALD). Various thicknesses of Al_2_O_3_ were achieved by changing the number of ALD cycles, and nine samples were prepared with the Al_2_O_3_ thicknesses ranging from 0.97 to 6.3 nm. Finally, for all the samples, 100-nm Al was evaporated onto the Al_2_O_3_ surface as the top contact through shadow masks, and back side contact was also formed through the evaporation of Al. The MIS structure is depicted in Figure 2a. Figure 2b is a cross-sectional transmission electron microscope (TEM) image of Al/Al_2_O_3_/SiC which presents that Al_2_O_3_ was uniformly deposited as a fully amorphous film.

In order to determine the generation of SiO_2_ and the content ratio of SiO_2_ and SiC, the XPS method is used. XPS experiments were carried out on a RBD-upgraded PHI-5000C ESCA system (PerkinElmer, Waltham, MA, USA) with Mg Kα radiation (*hν* = 1,253.6 eV), and the base pressure of the analyzer chamber was about 5 × 10^−8^ Pa. Ar ion sputtering was performed to clean the sample in order to alleviate the influence of carbon element in the air. Samples were directly pressed to a self-supported disk (10 × 10 mm) and mounted on a sample holder, then transferred into the analyzer chamber. The whole spectra (0 to 1,100 eV) and the narrow spectra of Si 2*p*, O 1*s*, C 1*s*, and Al 2*p* with much high resolution were both recorded, and binding energies were calibrated using the containment carbon (C 1*s* = 284.6 eV). Since the XPS spectra obtained consist of numerous overlapping peaks, curve fitting is necessary to separate the peaks from each other. The binding energies for the species were all correlated to the binding energies determined from standards in the handbook of XPS [17] and earlier studies [18,19]. These standards were also used to determine the full width at half-maximum (FWHM) and band type for curve fitting of multicomponent spectra, and it was found that the Gaussian distribution was the best model. Background removal was adopted according to the Shirley model and performed prior to curve fitting.

## Results and discussion

**Figure 3:**
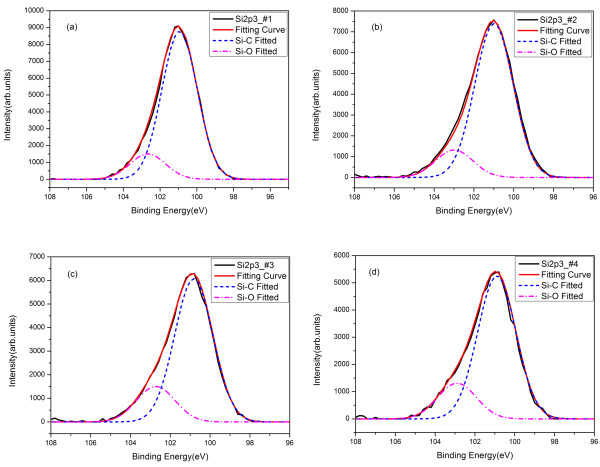
**Si 2*p *XPS spectra of samples 1, 2, 3, and 4 with varying thicknesses.** (**a**) Sample 1 with Al_2_O_3_ thickness of 1.3 nm. (**b**) Sample 2 with Al_2_O_3_ thickness of 1.98 nm. (**c**) Sample 3 with Al_2_O_3_ thickness of 2.32 nm. (**d**) Sample 4 with Al_2_O_3_ thickness of 3.59 nm. The black solid line represents the original data of Si 2*p* spectrum; the red solid line is for the fitting curve. The blue dash line stands for the Gaussian peak of Si-C bonds and the magenta dash-dot line stands for the Gaussian peak of Si-O bonds. Both Gaussian peaks were separated from the core-level Si 2*p* spectrum.

**Figure 4:**
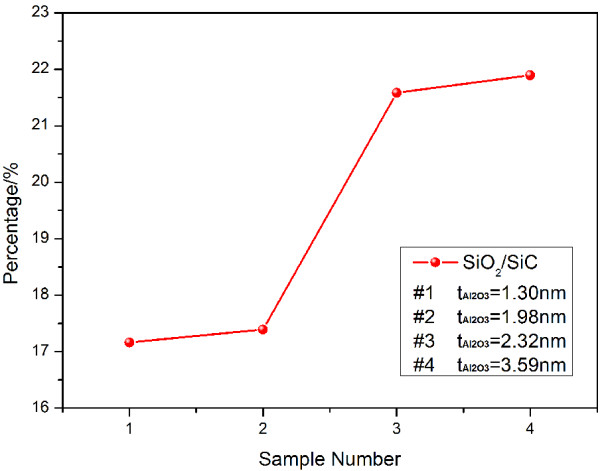
**The four samples’ content ratio of SiO**_**2 **_**and SiC.** The content ratio transfers to the area ratio of Si-O bond’s fitting curve and Si-C bond’s fitting curve.

Figure 3 describes the Si 2*p*3 core-level spectra of the four samples with the Al_2_O_3_ thicknesses of 1.3, 1.98, 2.79, and 3.59 nm, respectively. It is clear that the Si 2*p*3 spectrum can be fitted with two Gaussian peaks which correspond to Si-C bonds (100.9 eV, FWHM = 2.27 eV) and Si-O bonds (102.8 eV, FWHM = 2.27 eV). As illustrated in Figure 3a,b,c,d, all the Si 2*p*3 spectrum samples have a Si-C peak which associates with SiC from the substrate. Si-O species indicates that SiO_2_ exists at the Al_2_O_3_/SiC interface. This SiO_2_ is probably generated from SiC-heated substrate oxidized by Al_2_O_3_ since all the samples have been completely cleaned before the ALD process. Figure 4 demonstrates the evolution in the content ratio of SiO_2_ and SiC which is calculated by using the area of Gaussian fitting curve of the Si-O bond divided by the area of Gaussian fitting curve of the Si-C bond. It clearly and deliberately shows that the content of SiO_2_ oxidized by Al_2_O_3_ reaches an increase at the Al_2_O_3_ thickness of 1.98 nm. The content ratio of SiO_2_/SiC stays nearly at 17% in the Al_2_O_3_ film with the thickness beyond 1.98 nm. However, the content ratio of SiO_2_/SiC increases to 21.58% at the Al_2_O_3_ thickness of 2.32 nm and almost remains around 21.89% at the Al_2_O_3_ thickness of 3.59 nm and thicker samples. The content ratio of SiO_2_/SiC rises by about 24% from the 1.98-nm sample to the 2.32-nm sample, which is possibly due to the fact that the well-oxidized SiO_2_ begins to generate when the Al_2_O_3_ thickness is thicker than 1.98 nm.

**Figure 5:**
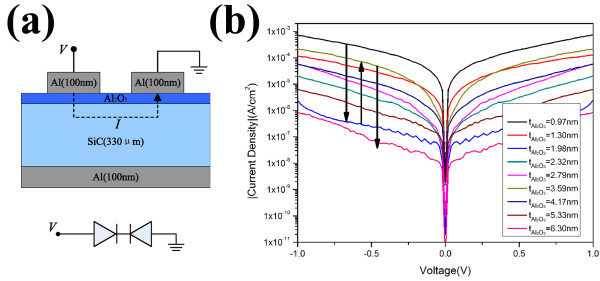
**Illustration of the back-to-back diode measurement setup and back-to-back Al/Al**_**2**_**O**_**3**_**/SiC diode measurements.** (**a**) Illustration of the back-to-back diode measurement setup where only the reverse current is measured. (**b**) Back-to-back Al/Al_2_O_3_/SiC diode measurements demonstrating the effective modulation of current density by the thickness of Al_2_O_3_.

The *I*-*V* characteristics of the Al/Al_2_O_3_/SiC MIS structure were measured by the circuit connections of the back-to-back Schottky diode as illustrated in Figure 5a. One advantage of the back-to-back diode measurement is that the large resistance contributed from the series resistance and the large resistance caused by the substrate can be eliminated. Another advantage is that both in positive and negative biasing, only the reverse current is measured, and fortunately, the change in reverse saturation current reflects the characteristic of the contact where maximum reverse saturation current is desired for ohmic contacts.

Figure 5b shows the *I*-*V* characteristics of an Al/ Al_2_O_3_/SiC diode with different thicknesses of Al_2_O_3_. Reverse bias current first decreases due to the increase of Al_2_O_3_ thickness which can block off the current and then has its minimum at the thickness of 1.98 nm which is suitable for the Schottky contact. When keeping on increasing the thickness, the reverse current rises since the formation of positive dipole between Al_2_O_3_ and SiO_2_ pulls down the SBH, and then, the reverse current reaches its maximum at the thickness of 3.59 nm which is suitable for ohmic contact. Next, the reverse current decreases as Al_2_O_3_ thickness increases owing to the large tunnel barrier induced by the thick Al_2_O_3_ film. The experimental *I*-*V* characteristics clearly indicate that current density is effectively modulated with the insulator’s thickness.

**Figure 6:**
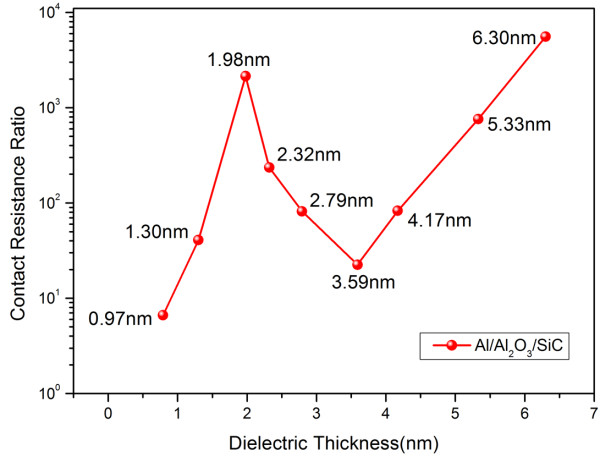
**Schematic of *R***_**C **_**versus *t***_**ox **_**for MIS contact by inserting Al**_**2**_**O**_**3**_**.***R*_C_ ratios are taken relative to the Schottky diode case.

Contact resistance (*R*_C_) of the Al/Al_2_O_3_/SiC MIS structure was further evaluated through contact end resistance method [20]. *R*_C_ involves two resistances in a series: a tunneling resistance (*R*_T_) due to the insulator and a resistance (*R*_SB_) caused by the Schottky barrier. When the thickness of Al_2_O_3_ is thinner than 1.98 nm, the dipole was not completely formed, and as a result, the inserted insulator blocks the current. In this range, along with the increase of the insulator, the contact resistance increases. According to the XPS result discussed above, the electronic dielectric dipole begins to create at the thickness of 1.98 nm. The formation of the dipole at the interface reduces the tunneling barrier and then raises the current across the contact in a reasonable region. Figure 6 shows the *R*_C_ versus the thickness of Al_2_O_3_, which provided that the contact resistance is modulated by the thickness of the insulator. It is interesting to find that there exists a trough because of the trade-off between a reduced barrier by the electronic dielectric dipole and an increased tunneling resistance by the accretion of the insulator’s thickness.

## Conclusions

In this work, we successfully realize the modulation of current density at the metal/SiC contact by inserting a thin Al_2_O_3_ layer between the metal and semiconductor. By varying the thickness of Al_2_O_3_, we can acquire the ideal current density and contact resistance based on our demands and achieve a transfer between Schottky contact and ohmic contact. The mechanism appears to be the coaction of a positive dielectric dipole decreasing the barrier and the tunneling resistance increasing the barrier. Consequently, this is a promising method to increase the performance of SiC electronic applications.

## Competing interests

The authors declare that they have no competing interests.

## Authors’ contributions

SZ carried out the sample fabrication and drafted the manuscript. WY carried out the device measurements. PZ and HL participated in the manuscript writing and results discussion. QS and DZ participated in the design of the study and performed the statistical analysis. All authors read and approved the final manuscript.
